# Editorial Gleanings

**Published:** 1854-10

**Authors:** 


### EDITORIAL.

### EDITORIAL GLEANINGS.

*Yellow Fever at Savannah.*—The epidemic at Savannah has been unusually severe; and the members of onr profession have suffered in a ratio fully equal to that of any other class. We learn that Drs. Schley, Wells, Ellis, Gordon, Harris and Wildman, have all fallen victims. The two last named in the list had only a short time previous to their death published statements strongly recommending the Muriated Tincture of Iron, as a remedy of great value in the treatment of Yellow Fever. Concerning it Dr. Wildman uses the following strong language, viz.:

"I have treated over one hundred and fifty cases of Yellow Fever since the 21st ult., (August), and of that number not one has died who commenced this remedy prior to Black Vomit. Since the 21st ult., I have not administered five doses of any other medicine. I give the tincture in doses varying from 20 to 60 drops every two hours in a tablespoonful of water for adults; and smaller doses for children. The cure is generally perfected in three days. This preparation of iron acts by medicating the blood and exerting styptic qualities upon the coats of the stomach."

It may be a very valuable remedy, but it appears not to have saved those who had the most implicit confidence in its virtues. But while the resident physicians are falling rapidly one after another, other members of the profession do not hesitate to take their places. Drs. Bacon and Brantley went into the city and offered their services in the midst of the epidemic. Such conduct evinces a nobler *heroism than that of Napoleon or Alexander the Great.*

---

*Fractures of the Humerus.*—Prof. F. H. Hamilton, of Buffalo recently read a paper before the Erie county Medical Society, in which he recommends Fractures of the Humerus to be dressed with the *arm straight* and a *long splint fitted* to its *whole length.*

The object is to prevent motion at the elbow-joint, which also moves more or less the fragments of bone and adds to the risk of leaving non-union.

---

*Fissures of the Rectum.*—Both Sir Benj. Brodie and Mr. Quain, claim that Fissures in the rectum can be completely cured by simply dividing ahe skin and mucous membrane without laying open the sphincter ani. This is a very desirable modification of the operation if it proves generally successful.

---

*Treatment of Cancer.*—Both Professor Arnott, of London and Bennet of Edinburgh, recommend the treatment of scirrhous tumours of the breast by the application of intense cold. A mixture of pounded ice and salt is to be applied daily. five minutes at a time.

It is said to remove the pain and cause the tumor to decrease very much in size. It is worthy of fair trial.

---

*Anti-Periodics.*—Dr. Bailey, of Emmettsville, Indiana, in his inaugural thesis published in the Western Lancet, recommends *Nitric Acid* as an efficient anti-periodic for the cure of intermittent fever.

He gives it in doses of from five to eight drops diluted in water, and repeated every six hours, without reference to the febrile exacerbation.

---

*Efficiency of Vaccination.*—Dr. Seaton, in a report recently read to the Epidemiological Society of Great Britain, gives the following valuable statistics, viz.:

"(A.) Table showing the average of deaths from small-pox out of every 1000 deaths from all causes, within the bills of mortality, for decennial periods, during the last half of the last century, (the half century preceding vaccination):—

| | | | | |
|---|---|---|---|---|
| For the 10 years ending | 1760 | 100 | in every | 1000 |
| " " | 1770 | 108 | " | " |
| " " | 1780 | 98 | " | " |
| " " | 1790 | 87 | " | " |
| " " | 1800 | 88 | " | " |

"(B.) Table showing the average of deaths from small-pox out of every 1000 deaths from all causes, within the bills of mortality, for decennial periods, during the first half of the present century (the half century succeeding the introduction of vaccination).

| | | | | |
|---|---|---|---|---|
| For the 10 years ending | 1810 | 64 | in every | 1000 |
| " " | 1820 | 42 | | " |
| " " | 1830 | 32 | | " |
| " " | 1840 | 23 | | " |
| " " | 1850 | 16 | | " |

These facts demonstrate very clearly the safety and value of the grand discovery of Dr. Jenner.

---

*Quack Remedies.*—At the late meeting of the American Pharmaceutical Association, the following suggestions were adopted concerning a subject of some importance, viz.:

"That the desire for medicine can be gratified in a legitimate way by regular officinal preparations;

That it is the duty, as well as interest, of the apothecaries and druggists, to advocate the use of the officinal preparations in lieu of the quackery of the day;

That it is the rightful interest of the regular pharmaceutists to divert the thousands, which now annually flow into the coffers of quacks, into their own limited stores, where of right it belongs. This can only be done by a united and sustained action on the part of the pharmaceutists and druggists of the Union, by which they will practically refrain from the sale or advocation of secret medicines, and substitute regular officinal compounds for them, correctly labelled with name and directions for use.

This course should receive the sanction of physicians, as the only one likely to remedy the evil, as the tendency to take medicine *ad libitum* is a feature of the Anglo-saxon race, duly inherited by the American people, which, whatever may be its faults, is as much their nature as is the love of political and personal freedom."

---

*Narcotism by Opium.*—In the October number of the American Journal of Medical Sciences, Dr. W. R. Bullock, of Delaware, relates a case in which a man of intemperate habits, aged about 40 years, on retiring to bed at 10 P.M., was supposed to have taken over two ounces of Laudanum. His unusual breathing attracted the attention of his wife, and Dr. Bullock was called at 12 o'clock, two hours after the poison had been swallowed.

He describes the symptoms as follows, viz.: "Found him lying on his back on a truckle-bed; countenance palid and bathed in a cold prespiration; lips somewhat livid; eye-balls turned up beneath the lids, and fixed; pupils contracted and immoveable; head hot; insensibility to external impressions profound; respirations three to four per minute, jerking, and somewhat stertorous; extremities warm; pulse accelerated, full and hard.

The treatment consisted in venesection to the extent of 12 oz., the application of cold to the head, sinapisms to the extremeties, and an enema to move the bowels, before positive evidence of his having taken the laudanum was obtained. Then an unsuccessful attempt was made to introduce the stomach tube, for the purpose of removing the contents of that organ. The patient continuing to fail, by one of Kinne's electro-magnetic machines, currents of electricity were passed through the muscles of the chest, the abdomen, and the diaphragm, and the effect kept up continuously. Sinapisms were also kept applied over the spine. His respiration increased slightly in frequency, and his pulse remained good. About 4½ A M. the patient awoke, and spoke to those around him, and remained conscious about three quarters of an hour. During this time his respiration was more frequent, but sighing, and his pulse became full and hard. He swallowed some ammonia and cold water with difficulty, and the electricity was discontinued. After the length of time mentioned he broke out in a profuse sweat, and rapidly sunk into a stste of more profound unconsciousness and depression than at first. The application of electricity was again resumed, the currents being passed not only through the muscles of the chest and abdomen, but also along the medulla oblonga and spine. The greatest effect was produced when the current was passed in the direction of the sphrenic nerve. This treatment was kept up until 1½ P. M., when the patient again suddenly awoke to consciousness, and recognized those around him. His respiration, though still slow and sighing, was improved, and the pulse became stronger, while the head was very hot. Feeble momentary currents of electricity were now continued, cold water applied to the head, sinapisms continued over the spine and extremities, while cold water, ammonia, strong coffee, and beef tea were given immediately. The patient continued from this time conscious, and slowly recovered, having been in a state of profound depression and unconsciousness, except an interval of three quarters of an hour, during 14 hours. It was noticed throughout that the heart's action was much less diminished than the process of respiration.

During the past summer, I was called in consultation to see a case of extreme narcotism. A nursing child, aged about 9 months, had been affected with diarrhœa, and the father gave her, about 8 A. M., half a tea-spoonful of what he had purchased at a drug store for Paragoric or Camphorated Tincture of Opium. The vial was also labelled *Paragoric*, but on examination its contents were found to be *Laudanum*. In a short time after the medicine was taken the child exhibited signs of spasms, and rapidly sunk in a profound stupor. The father, alarmed at its appearance, took it in his arms to the office of Dr. G. B. Page, which was near by. Before the Doctor saw it, however, the child was already entirely unconscious, and incapable of swallowing. The face was pale, the pupils contracted, the pulse feeble, and the respiration extremely slow, being not more than from three to five inspirations per minute; and if allowed to remain 15 or 20 minutes at rest, the respiration would seem to cease, and the pulse become extremely feeble. Dr. Page being satisfied that the laudanum had nearly disappeared from the stomach by absorption, made no attempt to introduce the stomach tube or evacuate the stomach in any way. To keep up respiration, however, he directed every five minutes a stream of cold water upon the head and chest, keeping it wrapped during the intervals in warm blankets. The cold invariably quickened the respirations for a time, and by its faithful repetition the child was apparently kept alive until I saw it at 4 P. M. It was then in the same condition as above described. I advised the dashings of cold water to be continued, and as nothing could be swallowed, I recommended the use of enemas of strong tea, repeated every half hour. This was done, and about 5 P. M. the child began to manifest signs of consciousness, and from that time rapidly recovered. There was a slight degree of febrile reaction and heat of head after the return of sensibility, but none before.

---

*Precocious Infant.*—In Morland's Extracts from the Boston Society for Medical Improvement, published in the American Journal of Medical Sciences for Oct. 1854, Dr. Blake relates the following case: "May 11th, attended Mrs. H., in her fourth confinement. After a tedious, though not severe labor, she was delivered of a female child, unnsually vigorous, and weighing at birth, eleven and a half pounds. On the third day subsequent, while visiting his patient, the nurse asked, 'Doctor, do you see our baby laugh?" Incredulous he crossed the room—but to his surprise, the infant returned an answering smile to the caresses of the nurse. With a look of intelligence, it turned to the father who stood over it as he patted its cheeks, and as quickly turned its eyes on Dr. B., when he did the same; and when he stepped aside, its gaze followed him (attracted, he supposes, by his spectacles) until, being out of view, it cried aloud; returning to its side, a smile of pleasure again greeted him, and the cry was once more repeated when he ceased to notice the little one. There was unmistakable evidence of pleasurable and sad emotions; and showing the distinct recognition of sensible objects, by an infant as yet scarcely sixty hours old. That it was no accidental circumstance the like manifestations, which were daily noticed afterwards, sufficiently prove."

---

*Powerful Effects of Small Doses of Opium.*—Dr. Morland relates a case on which a man on going to bed at 10 P. M., swallowed by mistake, a pill containing *five* grains of solid opium. The next morning he "was found lying on his left side breathing with an occasional snore; he was bathed with perspiration, which, increased by warm coverings, had soaked his night-flannel and shirt completely, had wetted the sheets and pillow-case, and even the ticking of the bed."

Every effort to arouse him produced but slight effect. An emetic of salt and water was given, and though vomiting was induced, he was aroused only momentarily. Strong coffee was administered with no better success. Injections of soap and water were administered, and a purgative of calomel, jalap, and ipecac. This not operating, and no change being induced after waiting some hours, 1½ drops of Croton oil were administered. This was followed by a moderate fecal discharge in about three quarters of an hour, and also a free flow of urine. He continued, however, soporose, pale, with pulse 68 or 70, and feeble until evening, when he rather suddenly became wakeful and restless. From that time he gradually recovered his usual health. Speaking of the small amount of opium which will sometimes produce dangerous effects, Dr. Christison records a case in which "four and a half grains of opium, combined with nine grains of camphor, given to an adult, proved fatal in nine hours." And Dr. J. B. S. Jackson has reported a case in which *five drops of laudanum* injected into the rectum of a child 18 months old, proved fatal in *six hours*. It will be seen from the foregoing that fatal results have followed the exhibition of smaller quantities of opium than some physicians recommend to be freely given in the treatment of dysenteries and fevers. It must be borne in mind, however, that the capability to bear with impunity any given quantity of opium depends very much on the condition of the patient. Thus, the quantity of opium which the same child would receive without any serious effects while laboring under an active dysentery, would prove speedily fatal if given during the existence of a bronchial or pneumonic inflammation.

---

*Epilepsy Treated by Ligation of Carotid*.—In the American Journal of Medical Sciences for Oct. 1854, Dr. Joseph B. Brown of the U. S. Army, relates a case of epilepsy of several years duration and of great severity, which was cured by trying the common carotid of one side. The patient was a female, aged 22 years, and had suffered a convulsive paroxysm every hour in the twenty-four ; yet she retained all her mental faculties perfect, and no cause could be detected adequate to account for the existence of the disease except the disordered circulation of blood in the brain. Dr. B. ligated the common carotid on the right side above the omohyoides muscle. No bad symptoms followed, and the patient has had no paroxysms of epilepsy since, a period of five years having now elapsed. No chloroform was used during the operation. D.

